# Time to tuberculosis development and its predictors among HIV-positive patients: A retrospective cohort study

**DOI:** 10.1371/journal.pone.0298021

**Published:** 2024-02-12

**Authors:** Abraham Teka Ajema, Yilkal Simachew, Meiraf Daniel Meshesha, Taye Gari

**Affiliations:** 1 School of Public Health, College of Medicine and Health Sciences, Hawassa University, Hawassa, Ethiopia; 2 Department of Internal Medicine, Dilla University, Dilla, Ethiopia; Clinton Health Access Initiative, SOUTH AFRICA

## Abstract

**Objectives:**

To assess the incidence and predictors of time to Tuberculosis (TB) development among Human Immunodeficiency Virus (HIV) positive patients attending follow-up care in health facilities of Hawassa, Ethiopia.

**Methods:**

We conducted a retrospective cohort study from April 1–30, 2023. A total of 422 participants were selected using a simple random sampling method. Data was collected from the medical records of patients enrolled between January 1, 2018 –December 31, 2022, using the Kobo toolbox. We used Statistical Package for Social Studies (SPSS) version 26.0 for data analysis. To estimate the duration of TB-free survival, we applied the Kaplan-Meier survival function and fitted Cox proportional hazard models to identify the predictors of time to TB development. Adjusted hazard ratios (AHR) with 95% confidence intervals were calculated and statistical significance was declared at a P-value of 0.05.

**Results:**

The overall incidence rate of TB among HIV-positive patients was 6.26 (95% CI: 4.79–8.17) per 100 person-years (PYs). Patients who did not complete TB Preventive Therapy (TPT) were more likely to have TB than those who did (AHR = 6.2, 95% CI: 2.34–16.34). In comparison to those who began antiretroviral therapy (ART) within a week, those who began after a week of linkage had a lower risk of TB development (AHR = 0.44, 95% CI: 0.21–0.89). Patients who received ART for six to twelve months (AHR = 0.18, 95% CI: 0.05–0.61) and for twelve months or longer (AHR = 0.004, 95% CI: 0.001–0.02) exhibited a decreased risk of TB development in comparison to those who had ART for less than six months.

**Conclusion:**

The incidence of TB among HIV-positive patients is still high. To alleviate this burden, special attention should be given to regimen optimization and provision of adherence support for better completion of TPT, sufficient patient preparation, thorough clinical evaluation for major (Opportunistic Infections) OIs prior to starting ART, and ensuring retention on ART.

## Introduction

Tuberculosis (TB) is one of the major infectious diseases in the world. It is the leading cause of morbidity and mortality worldwide, with a special predilection for certain high-risk population groups, such as those with HIV infection [1, 2].

Globally, an estimated 10.6 million incident TB cases, 1.4 million deaths among HIV-negative people and 187,000 deaths among HIV-positive people, for a combined total of 1.6 million were reported in 2021. Among all incident cases of TB, 6.7% were people living with HIV, whereas in Africa, the proportion reaches up to 19.8%. Worldwide, the total number of deaths from TB in people who are HIV-positive was 185,721, with African countries contributing about 73% of the deaths [2].

Ethiopia is listed as one of the top 30 countries with high TB and TB/HIV burden country according to recent estimates published by WHO for the years 2021–2025 [3]. The estimated proportion of TB among HIV-positive patients was 5.2% in 2021. Furthermore, the national mortality from TB was 1.7 per 100,000 population among HIV-positive patients during similar years [2].

Joint efforts are implemented to decrease the burden of TB among HIV-positive patients and vice versa. The national TB and HIV control programs act cooperatively through the implementation of TB/HIV collaborative activities aiming to strengthen TB case finding, TB prevention and TB/HIV co-infection management through different innovative and systematic approaches [1, 4, 5]. Ethiopia has adopted multiple interventions in TB/HIV programs in recent years, including rapid ART initiation through the “test and Treat” strategy in 2017 and the optimized Dolutegravir-based ART regimens in 2019 [3, 5, 6]. Another essential TB program intervention implemented with a top priority was the provision of TPT for all HIV-positive patients for latent TB treatment [7, 8].

Despite different program interventions in HIV care and treatment, TB still poses a significant challenge to HIV-positive patients, and healthcare providers working in ART clinics and TB/HIV programs.

There were previously conducted studies, but the follow-up and study periods were before the key HIV treatment recommendations were implemented whose impacts have not yet been studied well in the country, making prior studies to have outdated evidence. This study tried to assess incidence and predictors of time to TB development among HIV-positive patients attending follow-up care in health facilities of Hawassa, Ethiopia, by introducing new variables, using a retrospective cohort design with the most recent follow-up period, and covering multiple facilities.

## Material and methods

### Study design, area, and period

During April 1–30, 2023, we carried out an institution-based retrospective cohort study on HIV-positive patients’ medical records who were undergoing follow-up care at health facilities in Hawassa City, Ethiopia. Hawassa city is located around 273 km south of Addis Ababa and is the capital city of Sidama regional state. There are ten health facilities providing ART services under Hawassa city, serving for the major share of Sidama regional number of HIV-positive patients on follow-up care.

### Population and eligibility

We used all HIV-positive patients aged 15 and more years, newly enrolled to HIV follow-up care from January 1, 2018, up to December 31, 2019, at health facilities in Hawassa City as a source population. Those transferred in from other facilities before starting ART were included in the study. Patients diagnosed with TB and/or initiated anti-TB with clinician decision at enrollment, charts with unavailable baseline and follow-up information or missing charts were excluded from the study. We followed patients who fulfilled eligibility criteria through routine service provision for three years up to December 31, 2022. This period was selected to capture the time of recent recommendations in the HIV and TB/HIV programs and to have the nearest follow-up period.

### Sample size determination

The sample size calculation was done by a double population proportion formula using an open Epi version 3.01 Stat Calc for cohort design. The statistical assumptions used were power 80% and 95% confidence interval. Additionally, 42% TB proportion among HIV-positive patients with a hemoglobin level of <11g/dl and 62% TB proportion among HIV-positive patients with a hemoglobin level of >11g/dl were obtained from a study done at health facilities of the Afar regional state, Ethiopia [9] and assumed an equal size for exposed and non-exposed. After adding 10% for incomplete or missing charts and a 1.5 design effect due to the sampling procedure followed, the final sample size was calculated to be 422.

### Sampling technique and procedure

A two-stage sampling technique was used. We randomly selected three out of the ten health facilities in the city that provide ART services in the first stage by a simple random sampling technique. The three health facilities are Hawassa University Comprehensive Specialized Hospital, Adare General Hospital and Hawassa Millennium Health Center. These were high case load ART service-providing facilities serving 6,429 HIV-positive patients, contributing around 95% of the total caseload for Hawassa city and 60% for the total of the Sidama region [10]. The total number of HIV-positive patients newly enrolled from January 1, 2018, up to December 31, 2019, enlisted by their medical record number for the three facilities. The calculated sample size was proportionally allocated for each facility. Finally, the required sample size was obtained using a simple random sampling technique, employing computer-generated numbers from each health facility during the second stage of sampling.

### Variables

The outcome variable was time to TB development. The exposure variables were sociodemographic (age, residence, sex, client referral site, occupation, and disclosure at enrollment), baseline clinical variables (presenting symptom, BMI, OI comorbidity, functional status, WHO stage, anemia, and TB screening status) and Follow-up, care, and treatment interventions (time to linkage, time to ART initiation, initiated ART regimen, duration of ART, adherence to ART, ART regimen change and TPT completion).

### Operational definition

**Time to TB development** was the time from enrollment dates to ART clinic to TB diagnosis and/or initiation of anti-TB medication by clinician decision during the follow-up period. **TB case** was an event ascertained when diagnosed with TB by approved diagnostic workups [1] and/or started anti-TB medication by clinician decision during the follow-up period. **Censored** were those patients who were lost, died, transferred out before developing TB or did not develop by the end of the follow-up period. **Time to linkage** was between dates of HIV-positive status identification and enrollment to the facility for ART follow-up care. **Time to ART initiation** was the time between enrollment dates to the facility for ART follow-up care and ART initiation. **OI comorbidity** was evidence of diagnosis and/or treatment for any WHO stage-defining opportunistic infectious (except TB) and non-infectious diseases during the follow-up period. **Duration of ART** was the total time calculated between dates of ART initiation and event occurrence or until censored. **TPT complete** were those patients with both start and end date of TPT available with a calculated time interval of 6–9 months for isoniazid only regimen or 3–4 months for isoniazid and rifapentine combination regimens. **Adherence to ART** was documented adherence level to ART medications at the first visit or at the first month after enrollment, whichever comes early, as Good (≥95% or < 2 doses missed per month or < 3 dose missed per 2 months), fair (85–94% or 3–5 doses missed per 30 doses or 3–9 doses of 60 doses), and poor (less than 85% or > 6 doses of 30 doses or > 9 dose of 60 doses) [11].

### Data collection technique and quality management

We developed a data extraction tool based on the national ART intake and follow-up forms used in ART clinics. With 5% of the sample size, we pretested it and made the necessary revisions. Data was collected by six trained nurses on national basic consolidated ART training with experience in ART care provision. A one-day orientation on the study’s objective, data collection tools and procedure were provided to the data collectors. All relevant data were extracted by reviewing paper-based individual patient charts, ART registers, and electronic medical records (EMR). An electronic Kobo toolbox-based platform was used for data collection. The extracted data was verified between papers and electronic-based medical records. The authors had no access to information that could identify individual participants during or after data collection. The electronic medical record system has frequent and regular data quality assurance in place, in case of discrepant data between the two, the electronic one was taken. A lost to follow-up and death were confirmed by reviewing medical records and/or appointment registers of adherence case managers. The most recent laboratory results were used as a baseline value. If there was no registered laboratory test at baseline, results available within the first month of enrollment were used. If two results were registered within the first month, the first one was taken. A daily based onsite monitoring was done on the progress of data collection by the principal investigator.

### Data entry and analysis

Data was exported from the Kobo toolbox as an Excel file and then imported to SPSS version 26.0, which we used for coding, cleaning, and analysis. We performed a descriptive statistic to summarize and present the data. We calculated the TB incidence per 100 PYs after computing the total duration of the follow-up period. We used the Kaplan–Meier survival function to estimate the mean survival time of TB and to compare survival curves between different categories of explanatory variables by using the log-rank test. The Cox proportional hazard model was used to identify predictors of time to TB development. We have done a bivariable Cox proportional hazard analysis initially and variables with < 0.2 P-values were entered into multivariable Cox proportional hazard model. We computed AHR with 95% confidence intervals, and we declared statistical significance at a P-value of < 0.05. Multicollinearity was checked using variance inflation factor (VIF) and cutoff point was VIF > 10 to have significant collinearity among predictors. We have made comparison of CHR and AHR between potential confounders and declared a variable confounding by using a more than 10% cutoff value difference between the two. A stratified analysis was conducted to check for the presence of effect modifiers by conducting crude and stratum specific HR. We examined the assumption of proportional hazards graphically by plotting the log [−log (survival function)] estimates against log time plots for each covariate and by using a global test based on Schoenfeld residuals for the final model.

### Ethical consideration

As this study involved the analysis of secondary data obtained from patient’s medical records, the need for participant’s consent was waived by the Institutional Review Board of Hawassa University College of Medicine and Health Science with an approval letter under reference number IRB/245/15. Formal permission letters for each study facility were obtained from the Hawassa City Administration Health Office and Hawassa University College of Medicine and Health Science, School of Public Health.

## Result

### Sociodemographic characteristics

From the 422 randomly selected charts, 393 (93%) were included in the study. The median (Interquartile range [IQR]) age of the participants was 32 (27–40) years, and 237 (60.3%) of participants were females. At the time of enrollment majority, 314 (81.2%), were urban residents, and 281 (73.9%) of them were identified as HIV-positive within the same health facility (Table 1).

**Table 1:** Sociodemographic characteristics of study participants, Hawassa, Ethiopia, January 1, 2018 –December 31, 2022.

Characteristics		Frequency	Percent
**Age in years (n = 393)**	15–29	144	36.6
	30–44	183	46.6
	45 and above	66	16.8
**Sex (n = 393)**	Female	237	60.3
	Male	156	39.7
**Occupation (n = 367)**	yes	245	66.8
	No	122	33.2
**Residence (n = 393)**	urban	319	81.2
	rural	74	18.8
**Client Referral (n = 380)**	Within Facility	281	73.9
	Outside of Facility	99	26.1
**Disclosure (n = 368)**	yes	304	82.6
	no	64	17.4

### Baseline clinical characteristics

Two hundred and thirteen (54.2%) were symptomatic at presentation, and 155 (39.4%) individuals (39.4%) screened positive for TB. The median (IQR) of the BMI was 20.55 (18–24) kg/m^2^ (Table 2).

**Table 2:** Baseline clinical characteristics of study participants, Hawassa, Ethiopia, January 1, 2018 –December 31, 2022.

Characteristics (n = 393)		Frequency	Percent
**Presenting Symptom**	Yes	213	54.2
	No	180	45.8
**Functional status**	Ambulatory	82	20.9
	bedridden	21	5.3
	Working	290	73.8
**BMI**^a^ **(kg/m**^**2**^**)**	<18.5	109	27.7
	> = 18.5	284	72.3
**WHO**^b^ **Stage**	Stage 1	196	49.9
	Stage 2	42	10.7
	Stage 3	69	17.6
	Stage 4	86	21.9
**TB Screen**	Positive	155	39.4
	Negative	238	60.6
**OI**^c^ **Comorbidity**	Yes	202	51.4
	No	191	48.6
**Anemia**	yes	92	23.4
	no	301	76.6

^a^BMI, body mass index;

^b^WHO, World Health Organization;

^c^OI, opportunistic infection

### Care, treatment intervention and follow-up characteristics

For 333 (84.5%) patients, the time taken to link them to ART clinics from the identification of their HIV-positive status was less than one week. Additionally, 310 patients (78.9%) had initiated ART within one week of being linked to ART clinics. For most patients, 287 (73%), the initial ART regimen was TDF+3TC+EFV. During the follow-up period, 293 (74.6%) completed full course of TPT (Table 3).

**Table 3:** Care, treatment intervention and follow-up characteristics of study participants, Hawassa, Ethiopia, January 1, 2018 –December 31, 2022.

Characteristics (n = 393)		Frequency	Percent
**TPT**^a^ **Completed**	Yes	293	74.6
	No	100	25.4
**Linkage time in week**	< 1 week	332	84.5
	1 week and more	61	15.5
**ART**^b^ **Initiation time in week**	< 1 week	310	78.9
	1 week and more	83	21.1
**Duration of ART**^b^ **in year**	< 1 year	99	25.2
	1–2 years	39	9.9
	2 years and more	255	64.9
**ART**^b^ **Regimen (Initial)**	AZT^c^+3TC^d^_EFV^e^	5	1.3
	TDF^f^+3TC+EFV	287	73.0
	TDF+3TC+DTG^g^	92	23.4
	Other^h^	9	2.3
**ART**^b^ **Adherence**	good	300	76.3
	fair	26	6.6
	poor	67	17.0
**ART**^b^ **Regimen change**	yes	234	59.5
	no	159	40.5

^a^TPT, TB preventive therapy;

^b^ART, antiretroviral therapy;

^c^AZT, zidovudine;

^d^3TC, lamivudine;

^e^EFV, efavirenz;

^f^TDF, tenofovir disoproxil fumarate;

^g^DTG, dolutegravir;

^h^other, AZT+3TC+EFV & ABC+3TC+EFV

### Incidence of TB among HIV-positive patients

During the 3-year cohort period, 393 HIV-positive patients were followed for a total of 862.68 person-years (PYs) time. Among all cases, 54 (13.7%) had developed TB during the following period. The overall incidence rate was 6.26 (95% CI: 4.79–8.17) per 100 PYs, with 78% of the cases occurring during the first year of the follow-up period.

The highest incidence rate of TB was found among patients who took ART for less than six months (231.8 per 100 PYs), followed by those who did not complete TPT (44 per 100 PYs). In contrast, the lowest incidence rate was among patients with no presenting symptom at enrollment (0.43 per 100 PYs) followed by those with TB screen negatives 0.83 per 100 PYs (Table 4).

**Table 4:** Incidence of TB among participants by study variables, Hawassa, Ethiopia, January 1, 2018—December 31, 2022.

Characteristics (n = 393)		Survival status		PYs^c^ observation	IDR^b^ (per 100 PYs)	95% CI^a^
		TB	Censored			
**Age in years**	15–29	20	124	298.41	6.70	4.32–10.39
	30–44	25	158	405.23	6.12	4.17–9.13
	45 and above	9	57	159.04	5.66	2.94–10.88
**Sex**	Female	27	210	550.82	4.90	3.36–7.15
	Male	27	129	311.86	8.70	5.93–12.62
**Occupation**	yes	31	214	550.95	5.62	3.96–8.00
	No	22	100	253.45	8.68	5.72–13.18
**Residence**	urban	41	278	705.59	5.81	4.28–7.89
	rural	13	61	157.09	8.28	4.81–14.25
**Client Referral**	Within Facility	42	239	606.71	6.92	5.11–9.37
	Outside of Facility	12	87	229.76	5.22	2.97–9.20
**Disclosure**	yes	48	256	672.46	7.13	5.38–9.72
	no	6	58	120.64	4.97	2.23–11.07
**Presenting Symptom**	Yes	52	161	400.65	12.98	9.89–17.03
	No	2	178	462.03	0.43	0.11–1.73
**Functional status**	working	19	271	716.21	2.65	1.69–4.15
	Ambulatory & Bedridden	35	68	146.46	23.89	17.16–33.28
**BMI**^d^ **(kg/m**^**2**^**)**	<18.5	32	77	200.94	15.92	11.26–22.52
	18.5 and more	22	262	661.74	3.32	2.19–5.05
**WHO**^e^ **Stage**	Stage 1 & 2	23	263	686.41	3.35	2.22–5.04
	Stage 3 & 4	31	76	176.27	17.59	12.37–25.00
**TB Screen**	Positive	49	106	261.96	18.70	14.14–24.75
	Negative	5	233	600.72	0.83	0.35–1.0
**OI**^f^ **Comorbidity**	Yes	49	153	387.74	12.63	9.55–16.72
	No	5	186	474.94	1.05	0.44–2.53
**Anemia**	yes	26	66	149.99	17.33	11.80–25.46
	no	28	273	712.68	3.92	2.71–5.69
**TPT**^g^ **Completed**	Yes	8	285	759.84	1.05	0.53–2.11
	No	46	54	102.84	44.73	33.50–59.72
**Linkage time**	< 1 week	37	295	746.04	4.96	3.59–6.84
	1 week and more	17	44	116.65	14.57	9.06–23.44
**ART**^h^ **Initiation time**	< 1 week	33	277	694.54	4.75	3.38–6.68
	1 week and more	21	62	168.14	12.48	8.14–19.15
**Duration of ART** ^h^	< 6 months	39	31	16.83	231.80	169.35–317.26
	6–12 months	5	24	21.81	22.92	9.54–55.07
	12 months and more	10	284	824.05	1.21	0.65–2.25
**Initial ART**^h^ **Regimen**	DTG^i^ Based	13	79	197.18	6.59	3.82–11.35
	Non-DTG^i^ Based	41	260	665.5	6.16	4.53–8.36
**ART**^h^ **Adherence**	good	35	265	733.89	4.77	3.42–6.64
	fair & poor	19	74	128.8	14.75	9.40–23.13
**ART**^h^ **Regimen change**	yes	39	195	597.19	6.53	4.77–8.94
	no	15	144	265.68	5.65	3.41–9.37

^a^CI, Confidence Interval;

^b^IDR, incidence density rate;

^c^PYs, person-years;

^d^BMI, body mass index;

^e^WHO, world health organization;

^f^OI, opportunistic infection;

^g^TPT, TB preventive therapy;

^h^ART, antiretroviral therapy;

^i^DTG, dolutegravir.

### Kaplan-Meier survival curve

**Fig 1:**
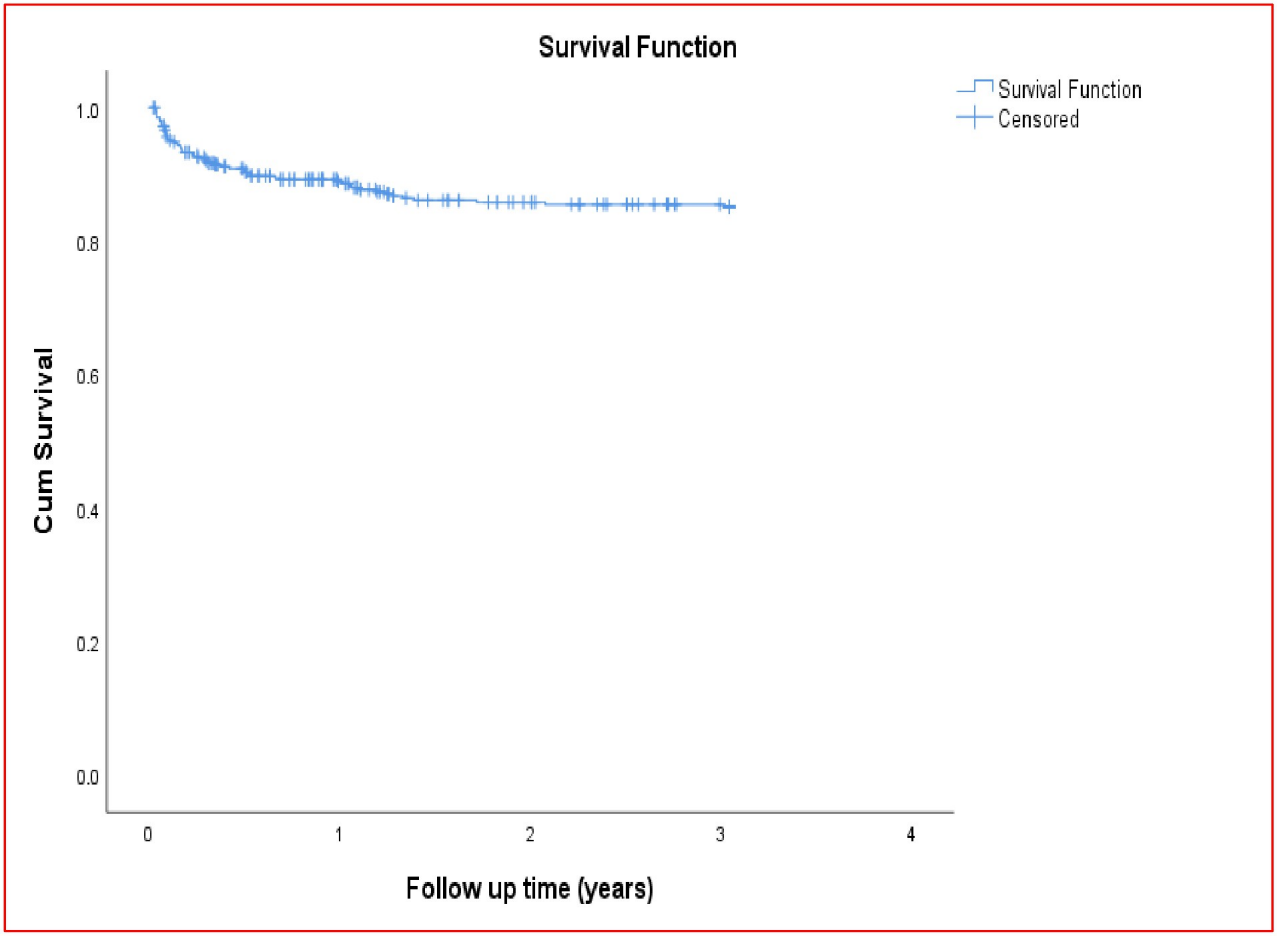
Kaplan—Meier survival estimate of study participants, Hawassa, Ethiopia, January 1, 2018—December 31, 2022.

**Fig 2:**
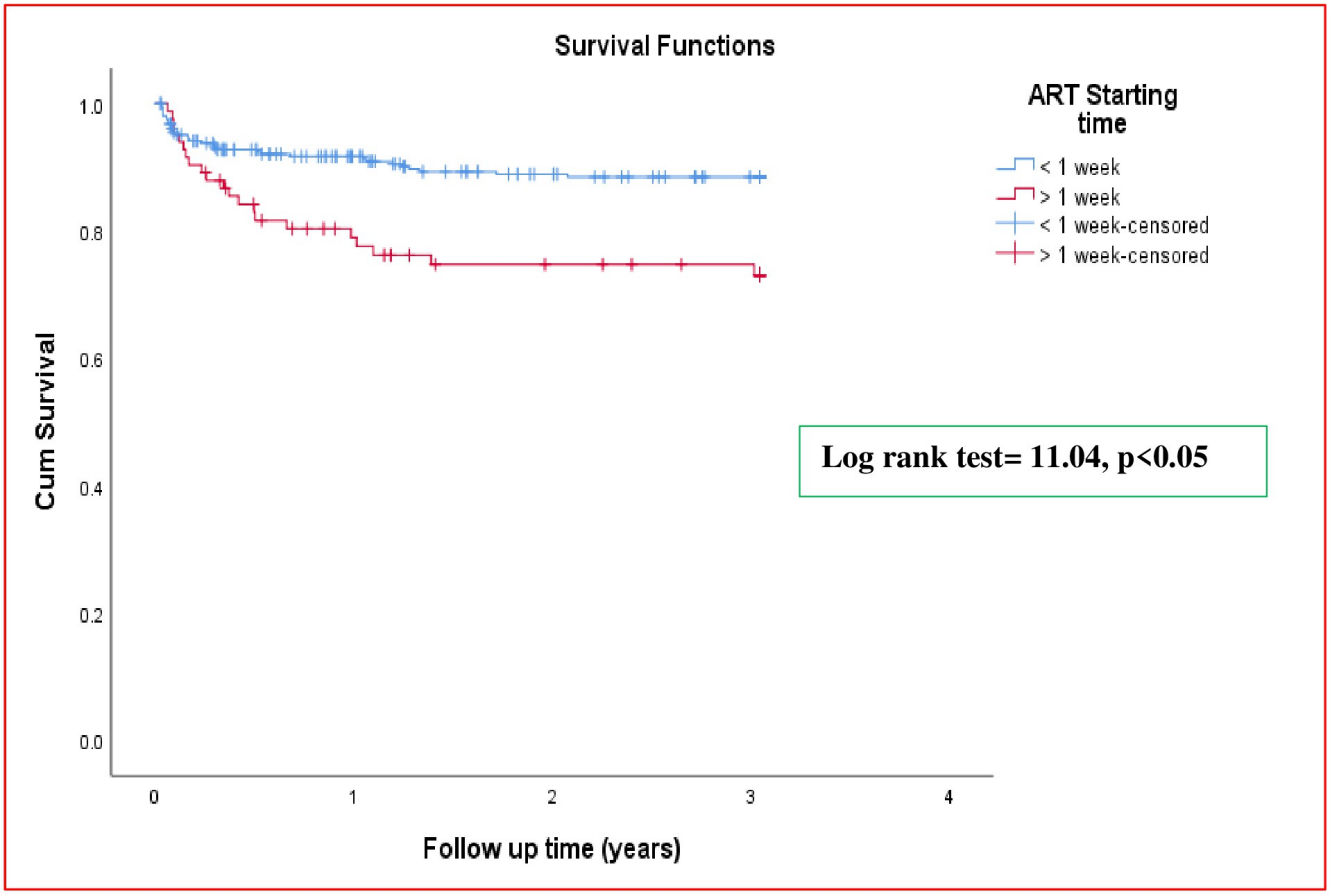
Kaplan—Meier survival estimate of study participants, by ART initiation time, Hawassa, Ethiopia, January 1, 2018—December 31, 2022.

**Fig 3:**
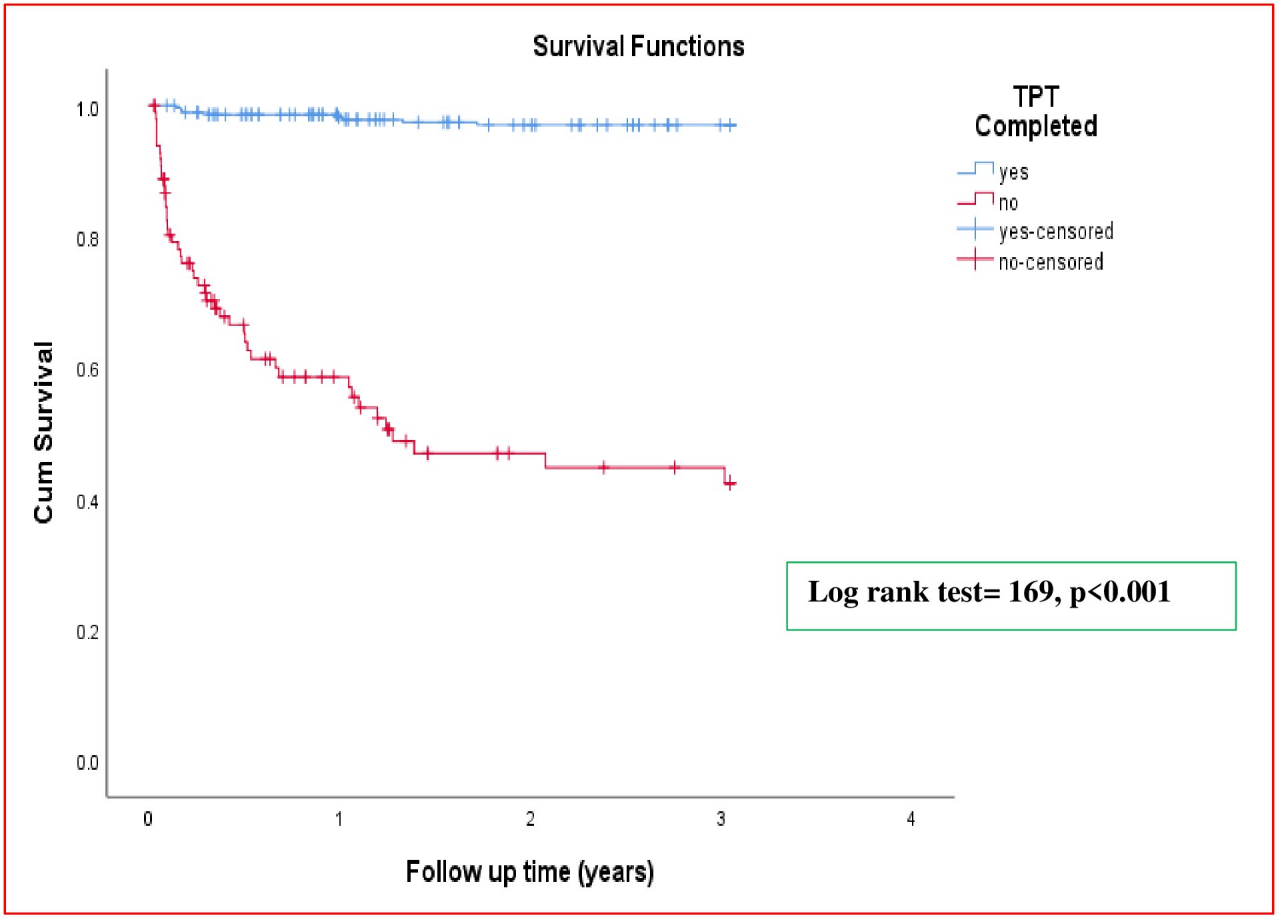
Kaplan—Meier survival estimate of study participants, by TPT completion, Hawassa, Ethiopia, January 1, 2018—December 31, 2022.

The mean survival time was 2.67 (95% CI: 2.58–2.77) years. The cumulative probability of TB survival at the end of the first, second, and third year of follow-up was 0.89, 0.86 and 0.85, respectively (Fig 1). Kaplan-Meier curve with log-rank test showed a statistically significant difference in the mean survival time of TB development between different categories of the explanatory variables (Figs 2 and 3).

### Predictors of time to TB development among HIV-positive patients

Among variables in the study, 12 gave a p-value of <0.2 in the bivariable Cox proportional hazard analysis. These variables were presenting symptoms, TB screening, baseline BMI, functional status, OI comorbidity, anemia, WHO clinical stage, TPT completed, ART initiation time, linkage time, ART adherence and duration of ART.

In the multivariable Cox proportional hazard analysis, after controlling for confounding variables such as anemia, WHO stage, and functional status; TPT completion, duration of ART and ART initiation time were identified as an independent predictors of time to TB development. Evidently, patients initiated on ART after a week of being linked to ART clinics as compared to those who did within a week had a 56% lower risk of TB development (AHR = 0.44, 95% CI: 0.21–0.89). Those patients who did not complete TPT, as compared to those who did, had six times higher risk of developing TB (AHR = 6.2, 95% CI:2.34–16.34). Those patients who took ART for six to twelve months had an 82% (AHR = 0.18, 95% CI: 0.05–0.61) lower risk of developing TB than who did for less than six months (Table 5).

**Table 5:** Bivariable and multivariable Cox proportional hazard analysis of study participants, Hawassa, Ethiopia, January 1, 2018—December 31, 2022.

Characteristics (n = 393)		Survival status		CHR^c^ (95% CI^a^)	AHR^b^ (95% CI^a^)	p-value
		TB	Censored			
**Presenting Symptom**	Yes	52	161	1		
	No	2	178	0.038 (0.009–0.157)	0.26 (0.048–1.37)	0.11
**Functional status**	working	19	271	1		
	Ambulatory & Bedridden	35	68	6.99 (3.99–12.25)	0.93 (0.46–1.9)	0.846
**BMI**^d^ **(kg/m**^**2**^**)**	<18.5	32	77	1		
	>18.5	22	262	0.23 (0.14–0.4)	0.91 (0.48–1.76)	0.789
**WHO**^e^ **Stage**	Stage 1 & 2	23	263	1		
	Stage 3&4	31	76	4.25 (2.47–7.29)	0.71 (0.35–1.44)	0.338
**TB Screening**	Positive	49	106	1		
	Negative	5	233	0.05 (0.02–0.13)	0.35 (0.11–1.13)	0.079
**OI**^f^ **Comorbidity**	Yes	49	153	1		
	No	5	186	0.09 (0.04–0.24)	0.65 (0.22–1.9)	0.432
**Anemia**	yes	26	66	1		
	no	28	273	0.27 (0.16–0.47)	1.14 (0.62–2.1)	0.683
**TPT**^g^ **Completed**	Yes	8	285	1		
	No	46	54	28.89 (13.53–61.68)	6.2 (2.34–16.4)	<0.001
**Linkage time**	< 1 week	37	295	1		
	1 week and above	17	44	2.73 (1.54–4.86)	1.71 (0.86–3.38)	0.123
**ART**^h^ **Initiation time**	< 1 week	33	277	1		
	1 week and above	21	62	2.45 (1.42–4.24)	0.44 (0.21–0.89)	0.023
**Duration of ART** ^h^	< 6 months	39	31	1		
	6–12 months	5	24	0.11(0.04–0.29)	0.18 (0.05–0.61)	0.006
	12 months and more	10	284	0.002 (0.0003–0.007)	0.004 (0.001–0.02)	<0.001
**ART**^h^ **Adherence**	good	35	265	1		
	fair & poor	19	74	2.16 (1.23–3.79)	0.62 (0.31–1.24)	0.173

^a^CI, confidence interval;

^b^AHR, adjusted hazard ratio;

^c^CHR, crude hazard ratio;

^d^BMI, body mass index;

^e^WHO, world health organization;

^f^OI, opportunistic infection;

^g^TPT, TB preventive therapy;

^h^ART, antiretroviral therapy.

## Discussion

Our study’s findings showed an overall incidence of TB of 6.26 per 100 person-years throughout the three years of follow-up, indicating that among HIV-positive patients, TB continues to be one of the significant OI diagnosed. TPT, ART start-up time, and ART duration were also found in the study to be statistically significant independent predictors of when TB will develop.

The incidence of TB among HIV-positive patients in this study was comparable with retrospective cohort studies previously done in Ethiopia Arbaminch (5.36), Addis Ababa (6.82) and Gondar (7.88) per 100 PYs [12–14]. However, ours was higher than the South African prospective cohort study (2.44 per 100 PYs) [15]. This lower incidence could be due to the fact that acid fast bacilli (AFB) in sputum or tissue was used as a diagnostic modality for TB in the South African study, which is known to have a lower detection rate among HIV-positive patients [16]. On the contrary, the incidence was lower than study conducted in India (24.3 per 100 PYs) [17] and in Tanzania (20.8 per 100 PYs) [18]. This difference might be due to the underlying higher burden of TB and HIV in these geographic areas. In another retrospective cohort study done in the Afar region, north-east Ethiopia (8.64) [9] and in Hawassa city, southern Ethiopia (8.79) [19] per 100 PYs were reported. Since the follow-up time for the above studies was more than ten years prior to the present one, multiple interventions were not being used; these higher rates may be justified.

Concerning the timing of TB development, our study is consistent with others as most TB cases occurred during the first year of follow-up time after enrollment to care [9, 20, 21]. Such consistency could be related to late diagnosis of HIV-positive status. When patients newly diagnosed present to care late with OIs, they tend to be identified earlier during their chronic care follow-up [22]. The other reason for this could be that the first six months after ART initiation are critical for a clinical phenomenon called IRIS (Immune Reconstitution Inflammatory Syndrome), whereby paradoxical or unmasked OIs tend to occur depending on the baseline immunological status of the patient and extent of baseline OI screening and diagnosis [23].

TB Preventive Therapy as a protective factor was consistent with studies conducted in Addis Ababa (5 times) [12] and Debre Markos study (4 times) [24] increased risk of TB development when not taken. Such consistency in TPT could be related with TPT being the mainstay of TB preventive strategies in treating latent TB infection, hence preventing reactivation of TB [8].

Another comparable finding regarding the duration of ART was with an Ethiopian meta-analysis; the pooled incidence of TB per 100 PYs among patients on ART for 60 or less months was 5.1 (95% CI: 3.64–6.46) compared with those with 60 months or more TB pooled TB incidence of 4.1 (95% CI: 1.87–6.27) [20]. The risk of TB development during follow-up is decreased with longer ART duration. This could be due to the benefit of ART-related immune recovery with resultant improved competency of the immune system in defending against the development of OIs.

A contradicting finding regarding the timing of ART initiation was reported by prior studies as compared with ours. Rapid ART initiation, both on the same day and within seven days, reduced OIs like TB as reported in a randomized controlled trial [25]. In one meta-analysis, rapid ART initiation has been shown to lower the risk of TB compared to delayed initiation [26]. In the Southern Ethiopian cohort, TB incidence decreased by 75% after the implementation of rapid ART initiation as part of the ‘test and treat’ strategy compared to the delayed one [27]. The reasons for our different results could be explained after understanding that the main benefit of rapid ART initiation is that early viral suppression with its resultant benefits associated with immune recovery. Such benefits are determined by the type of started ART regimen, the clinical status of the patients, the readiness of patients for lifelong ART and patient preparation by providers through proper counselling and optimal opportunistic disease screening. When patients are started on Integrase Strand Transfer Inhibitors (ISTI) such as Dolutegravir-based regimens, are ready both clinically and psychologically for lifelong ART; the benefit of rapid ART initiation could be maximal [28]. Furthermore, evidence from Rwanda suggest that for HIV-positive patients newly presenting to care with advanced immune suppression when they are rapidly initiated on ART; they had fear for side effects of the drugs and the lifelong treatment with resultant poor medication adherence [29]. Therefore, individuals with delayed ART initiation could have had more time to process and accept their HIV-positive status. This additional time could have allowed them to overcome the initial shock and emotional distress associated with the diagnosis, leading to improved clinical, psychological, and emotional well-being with optimal medication adherence. As a result, these individuals may have been more motivated and prepared to commit to their treatment regimen, including regular medication adherence. Besides the adherence-related factor, when ART is initiated rapidly without proper OI screening from poor diagnostic set-up or missed opportunities by health care providers with a significant focus on the speed of ART initiation, it could not lead to achieving the observed viral suppression and immune recovery.

Although anemia is not found to be a significant predictor of TB in our multivariable Cox regression analysis, it had been reported as one in prior studies in the past [9]. It is one of the risk factors and/or outcomes of chronic infectious disease. However, in our study, we reviewed documented evidence of anemia and/or hematocrit measurement, which might not be accurately captured in the medical records. The ISTI-containing ART is the preferred regimen for improving viral suppression, hence immune recovery and prevention of OIs [23]. However, in our study, the significant effect of the ART regimen was not shown. This interpretation should be taken with caution, and further large-scale study might be essential to produce evidence for the future.

## Strength and limitations of the study

### Strength

The cohort nature of the study ensured temporality between exposure and outcome variables in the associations produced. As we have tried to analyze a time to event data by considering relevant baseline and follow-up information in the prediction of time to TB development among HIV-positive patients on routine chronic care follow-up.

### Limitations

Although the study tried to address its objectives from the available data sources, it was not free from limitations. The retrospective nature of the study has limited us in including all possible sociodemographic factors associated with TB, such as housing condition, family size, and household income were some of the plausible factors which could affect the conclusion drawn in this study. The CD4 count of the patients was not included in the study due to the test interruption during the follow-up periods which will cause for the lack of objective immune status measurement in the study. In addition, calibration or standardizations of scales and steps in measurements of weight and height were not ensured as it was ascertained by medical record review which might potentially be introducing an information bias in the collected data. The sample size could have been smaller for some categories of variables with wider confidence intervals where scientific evidence-based categorization was undertaken to make associations.

## Conclusion

The overall incidence of TB among HIV-positive patients was high. TPT completion, ART duration and ART initiation time were independent predictors of time to TB development. The health care providers and TB/HIV programs should prioritize TPT regimen optimization and provision of adherence support for better completion of TPT by ensuring better coverage of short-course medication implementation. Healthcare providers should give adequate time for patient adherence preparation and perform a thorough clinical evaluation for major OIs prior to ART initiation, and in ensuring retention on ART for lifelong care and treatment.

## Supporting information

S1 Dataset (SAV)
